# Draft Genome Sequence of *Rheinheimera* sp. KL1, Isolated from a Freshwater Lake in Southern Saskatchewan, Canada

**DOI:** 10.1128/genomeA.01177-15

**Published:** 2015-10-08

**Authors:** Brady R. W. O’Connor, Benjamin J. Perry, Christopher K. Yost

**Affiliations:** University of Regina, Regina, Saskatchewan, Canada

## Abstract

*Rheinheimera* sp. KL1 was isolated from an algal bloom in Katepwa Lake, Saskatchewan, Canada. The isolate shares genetic and physiological similarities with *Rheinheimera tangshanensis*. The genome is estimated to be 4,295,060 bp in length with a GC content of 46.37%. Sequence analysis suggests the strain carries a previously uncharacterized prophage.

## GENOME ANNOUNCEMENT

Strain KL1 was isolated in 2013 from Katepwa Lake (50.6833° N, 103.6167° W), which is situated along the Qu’appelle river system in southern Saskatchewan, Canada. The strain was isolated from an algal bloom sample taken during mid-summer. The strain is a Gram-negative, aerobic, motile, rod-shaped bacterium with nonpigmented colonies. Colonies grew in lysogeny broth (LB) and agar media, with growth between 0 and 2% NaCl but not 4%; optimal growth was observed at 0.5% NaCl. Strain KL1 grew within a pH range of 5.9 and 8.8, and the optimal temperature for growth was observed to be 30°C. Analysis of the 16s rRNA gene revealed that KL1 was most similar to *Rheinheimera tangshanensis* (1, 2). Evaluation of carbon catabolism in KL1 was conducted using the GN2 assay supplied by Biolog (Hayward, CA, USA), following the manufacturer’s specifications. The resultant profile of carbon source catabolism closely resembled that of *R. tangshanensis* (2). At present there are 19 proposed species of *Rheinheimera* in the NCBI taxonomic database*,* and all have been isolated from aquatic environments. Strain KL1 represents the seventh sequenced *Rheinheimera* genome and the first genome to be sequenced from a putative strain of *R. tangshanensis*.

Genomic DNA used for sequencing was extracted from bacteria grown on LB agar plates at 30°C. Library preparation was done using the Ion Plus fragment library kit. Sequencing was done on the Ion Torrent PGM using 400-bp sequencing chemistry. Sequencing resulted in 1,267,837 reads that were assembled using SPAdes version 3.5.0 (3). The final assembly of KL1 contained 48 contigs with an *N*_50_ value of 153,243 and an estimated genome coverage of 79×. The genome was uploaded to NCBI’s Prokaryotic Genome Annotation Pipeline for annotation.

The assembled genome of KL1 is 4,295,060 bp in size with a GC content of 46.37%. The genome is predicted to contain 3,930 genes with 42 tRNAs. The genome was analyzed using the Antibiotic Resistance Genes Database (ARGD) and PHAST database (4, 5). Analysis by ARGD predicted the presence of a putative *acrB* multidrug RND efflux pump associated with resistance to acriflavin, beta-lactams, and macrolides. PHAST server analysis identified a 37,078-bp intact prophage region within the KL1 genome. The putative prophage KLPP is predicted to have a GC content of 46.9% and contain 43 coding sequences, including all the necessary genes for phage assembly. BLAST analysis of the KLPP nucleotide sequence to the NCBI database did not result in any highly similar sequences. The most similar sequence observed, with 69% sequence identity, was found in a prophage region of *Tolumonas auensis* DSM9187*.*

### Nucleotide sequence accession numbers.

The draft genome of *Rheinheimera* KL1 has been deposited at DDBJ/EMBL/GenBank under the accession number LAMX00000000. The version described in this paper is the first version, LAMX01000000.
